# Supplementary figures and images for: Quantifying the economic effects of different fishery management regimes in two otherwise similar fisheries
Source: PLoS One. 2023 Jun 20;18(6):e0287250. doi: 10.1371/journal.pone.0287250 (PMC10281562; doi:10.1371/journal.pone.0287250)

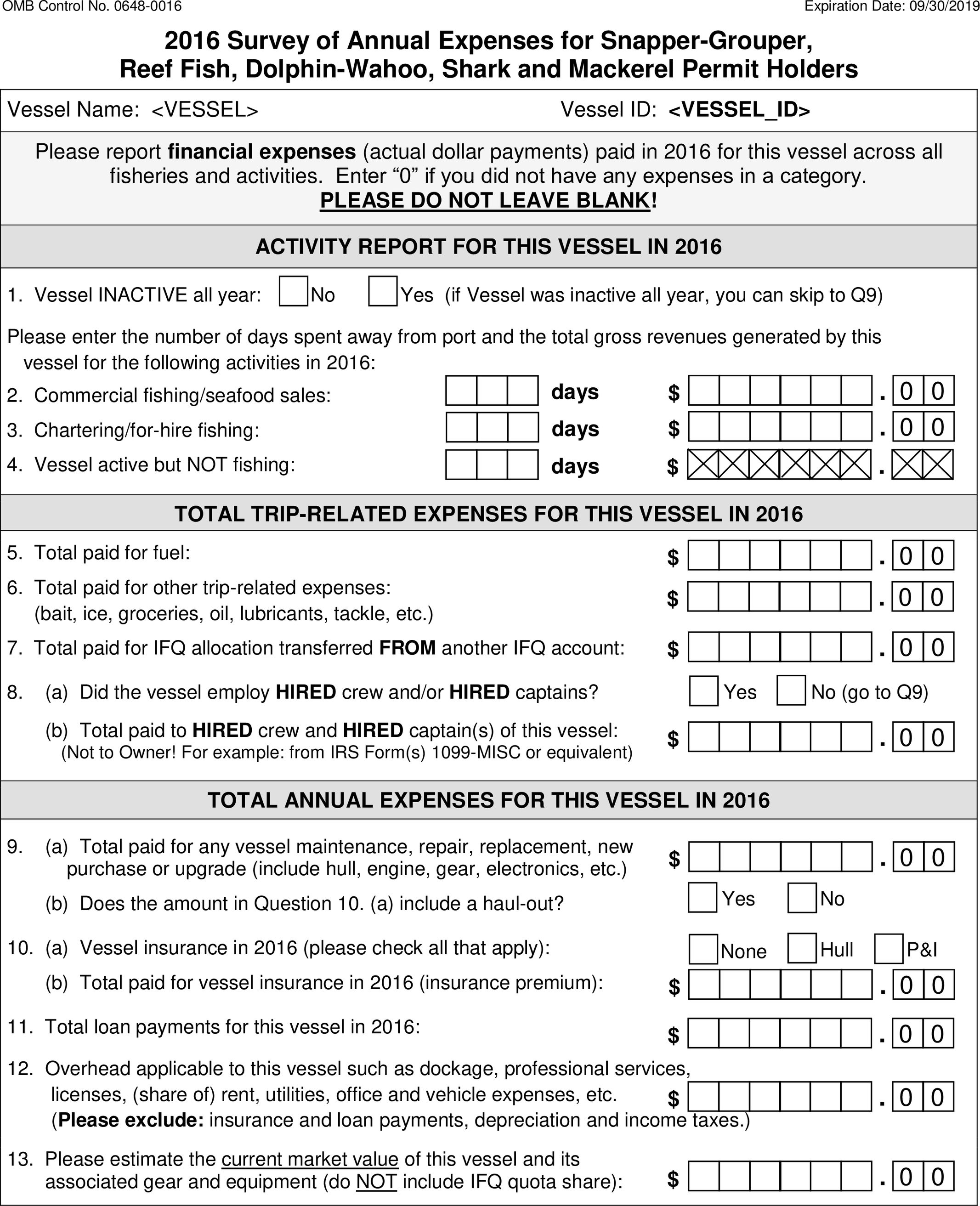

Supplement: S1 Fig — (TIF) [file pone.0287250.s001.tif]
