# Supplementary material for: Quantifying the economic effects of different fishery management regimes in two otherwise similar fisheries
Source: PLoS One. 2023 Jun 20;18(6):e0287250. doi: 10.1371/journal.pone.0287250 (PMC10281562; doi:10.1371/journal.pone.0287250)
Supplement: Attachment — Submitted filename: Response to Reviewers.docx [file pone.0287250.s002.docx]

Dear PLOS ONE editors,

Attached is a copy of the newly revised paper, “Quantifying the economic effects of different fishery management regimes in two otherwise similar fisheries” (Manuscript Number: PONE-D-22-30978). We believe we have addressed the concerns raised by the PLOS ONE editorial staff and the manuscript reviewers. Our response to the major comments are below.

PLOS ONE

“Please ensure that your manuscript meets PLOS ONE's style requirements, including those for file naming”

Done, and Figures have been moved into separate files.

“In your Data Availability statement, you have not specified where the minimal data set underlying the results described in your manuscript can be found. “

“We note that you have indicated that data from this study are available upon request. PLOS only allows data to be available upon request if there are legal or ethical restrictions on sharing data publicly.”

We added language into the document to this effect. Aggregated data and supporting documentation can be found at the NOAA repositories: <https://repository.library.noaa.gov/>.

“Please include your full ethics statement in the ‘Methods’ section of your manuscript file. In your statement, please include the full name of the IRB or ethics committee who approved or waived your study, as well as whether or not you obtained informed written or verbal consent. If consent was waived for your study, please include this information in your statement as well.”

Our surveys are reviewed by the US Office of Management and Budget, which is our IRB-type process that follows US federal law.

“We note that [Figure 1] in your submission contain [map/satellite] images which may be copyrighted. “

We have obtained and will upload the signed permission from the figure’s creator.

“Please review your reference list to ensure that it is complete and correct”

Done and revised according to PLOS ONE standards.

REVIEWER 1

“ the authors assume that these are preferable systems because they return more economic value from a renewable natural resource”

“Privatizing fishing resources through ITQ systems may result in higher rents, but they may also reduce the extent to which those fisheries distribute those rents through the wider economy”

“ as long as the authors mention the social costs that many studies have found with ITQ management systems, particularly regarding equity and impacts on crew vs. owner-operators.”

“Privatizing fishing resources through ITQ systems may result in higher rents, but they may also reduce the extent to which those fisheries distribute those rents through the wider economy”

Both reviewers noted that we stressed the importance of economic efficiency almost exclusively, and downplayed the importance of some of the negative impacts of ITQs. We acknowledge that and added language in the introduction and discussion sections (including a new paragraph) on some of the negative effects noted in the literature. We also make it clear that our focus is on the economic efficiency of management systems and should be received thusly.

“Note the 2021 Oceans Studies Board /NAS and Five Year Review of the Gulf tilefish/grouper ITQ program”

We added comments and cites accordingly.

“Their wording that “the SAT SG fishery only uses 29% more labor than the GOM RF fishery” deemphasizes this, but 29% is close to one-third of the labor”

A fair point and we replaced the phrase “relatively minor” with “lesser”,

REVIEWER 2

“question of what is the best approach is not primarily a scientific issue and in evaluating the options and potential solution it is important to know and understand the reasons for the current arrangements in both fisheries.”

“the choice of management approach is not simply a stark choice between one or the other. “

“L125 – 128. ‘Researchers have noted the effects, positive and sometimes negative of ITQs ….’ or something similar to make it clear that the papers referred to include both.”

“L50. The authors need to make it clear here and elsewhere that where they refer to ‘rationalizes’, ‘efficiency’ etc, they are referring only to economic rationalizing etc. The consequences of ITQs may not lead to rationalizing on other objectives – a well known example is the decline in fishing and hence rural populations in more remote regions of Norway as a result of ITQs.”

We added language acknowledging this, including a paragraph of some of the detrimental social effects of ITQs, as well as citations from both Councils regarding the management goals of the ITQs or Fishery Management Plans. See our comments to Reviewer 1. We also point out that the sole ITQ in the South Atlantic region (wreckfish) may provide a middle ground between the current management and the relatively unrestricted ITQ in the Gulf.

“ lines 356-357 it is stated that “Regulations for most of the major commercial fishing species in the SAT SG fishery are intended to extend fishing opportunities””

We provided more details and a citation on this in the conclusion after help from Council staff.

“L35. The term ‘traditional fisheries management’ used here and elsewhere needs to be qualified because what is traditional in the US is not necessarily traditional elsewhere. They could simply state something like ‘In traditional fisheries management in the USA (subsequently referred to as traditional fisheries management)”

L91. Should be ‘Some - ’ or ‘Many economists…’ Not all economists assume minimizing costs is the sole objective.

L215 and following. The authors should explain for both GOM and SAT how the total catch is regulated. Presumably in the GOM, a total allowable catch is set for each species or species assemblage, which is then divided into individual quotas according to the rights held by each holder. That, or if some other arrangement, should be explained. The likely approach in the SAT is less clear to readers unfamiliar with the fishery (including this one).

L269. Explain or define ‘IFQ leasing’.”

These minor edits were completed, thank you.

“L474 – 476. A reference or substantiation of some sort is needed for this statement.”

We edited the statement to better reflect our focus on the spread between returns in the two regions.

“L602-604. By omission these two sentences imply that discarding is not an issue in the GOM fishery. That would be very surprising as discarding and high-grading are problems in many other ITQ and IQ systems. The status in the GOM, in accordance with best available information, should also be stated here.”

Good point and we acknowledge that in the revised paper along with citations.

“L344. Is there an explanation for why the SAT SG fishers receive a lower price per pound than the GOM fishers? Is it a result of the seasonal effect referred to elsewhere or are there other factors too?”

We are uncertain to what extent seasons or other factors are causing this price difference, although we are aware of ongoing research investigating it right now.

“paper is too long and goes into too much detail in places”

It is definitely a long paper, but we wanted a thorough investigation of these issues, and chose PLOS ONE for its tolerance for somewhat long pieces with multiple graphics.
